# Polycyclic aromatic hydrocarbons (PAHs) in black crusts on stone monuments in Milan: detection, quantification, distributions, and source assessment

**DOI:** 10.1007/s11356-024-35134-4

**Published:** 2024-09-28

**Authors:** Maria Ricciardi, Antonio Faggiano, Antonino Fiorentino, Maurizio Carotenuto, Andrea Bergomi, Valeria Comite, Oriana Motta, Antonio Proto, Paola Fermo

**Affiliations:** 1 Dipartimento di Chimica e Biologia, University of Salerno, Via Giovanni Paolo II 132, 84084 Fisciano, SA Italy; 2 Consorzio Interuniversitario Nazionale per la Scienza e la Tecnologia dei Materiali (INSTM), 50121 Florence, Italy; 3 Dipartimento di Chimica, University of Milan, Via Golgi 19, 20133 Milan, Italy; 4 Dipartimento di Medicina, Chirurgia e Odontoiatria, University of Salerno, Via S. Allende, 84081 Baronissi, SA Italy

**Keywords:** Air pollutants, Polycyclic aromatic hydrocarbons, Black crusts, HPLC–DAD, Diagnostic ratios

## Abstract

In the field of conservation of cultural heritage, one must always consider the environmental conditions in which the works of art are located and the level of atmospheric pollution to which they are exposed, especially in the case of monuments stored outdoors. The present study is focused on the detection and the quantification of polycyclic aromatic hydrocarbons (PAHs) in black crust samples from the Monumental Cemetery of Milan (Italy), and the assessment of their sources through the analysis of the distributions of the different compounds in the samples, together with the use of diagnostic ratios. Six black crust samples taken from funerary monuments were analyzed. Fourteen polycyclic aromatic hydrocarbons were identified (naphthalene, acenaphthylene, acenaphthene, fluorene, phenanthrene, anthracene, fluoranthene, pyrene, chrysene, benzo[a]anthracene, benzo[b]fluoranthene, benzo[k]fluoranthene, benzo[a]pyrene, indeno[1,2,3-cd]pyrene) by high-performance liquid chromatography with a diode-array detector (HPLC–DAD), with a total concentration from 0.72 to 3.81 μg/g (mean of 1.87 μg/g). The known carcinogenic benzo[a]pyrene accounted for 5–10% of the total polycyclic aromatic hydrocarbons in the samples analyzed, with concentrations up to 0.20 μg/g. Moreover, the study of the distribution and diagnostic ratios allowed us to confirm that anthropogenic sources such as traffic and the proximity of the train station are the major causes of the degradation of the monuments contained in this Cemetery.

## Introduction

**Fig. 1:**
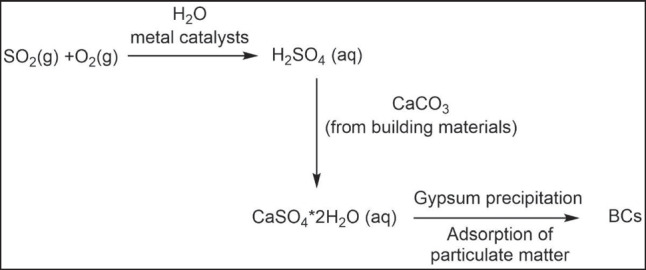
Schematic description of the reactions involved in the black crust formation

Air pollution is considered one of the main issues for the preservation of cultural heritage, especially when considering buildings and monuments located in very large outdoor areas and in cities highly exposed to anthropogenic pollution (Di Turo et al. 2016; Bergomi et al. 2023; Pironti et al. 2023). There are several air pollutants, especially acids such as CO_2_, NOx, and SOx, that can cause degradation of cultural heritage itself, one of the most dangerous being sulfur dioxide (SO_2_) (Pironti et al. 2020, 2021; Ricciardi et al. 2022b, 2024b; Faggiano et al. 2023). One of the principal building material deteriorations is the formation of soluble salts within porous materials as a consequence of dissolution–crystallization and hydration–dehydration cycles (Flatt 2002). NOx can react with water contained in the porous building materials to form acids and/or nitrite/nitrate ions that can act as agents of corrosion and degradation of materials, and in addition, NOx enhances the adsorption of SO_2_ in stones. Indeed, following several chemical reactions occurring in the atmosphere, SO_2_, is oxidized to H_2_SO_4_ which, due to its acidic character, can attack the surfaces of historical monuments and buildings by chemically interacting with the material of which they are made (generally a carbonate matrix, CaCO_3_). These reactions occur in the presence of other atmospheric pollutants such as particulate matter, which contain heavy metals that seem to be involved as catalysts in the sulphation process, ultimately leading to the formation of gypsum (CaSO_4_•2H_2_O). Sulfation process consists of the formation of sulfuric acid from wet atmospheric sulfur dioxide and its interaction with calcium carbonate (the stone substrate of the monument) to form gypsum. Each step of this process can be favored by several factors, including the presence of various heavy metals adsorbed on carbonaceous particles, the role of which is still unclear (Comite et al. 2023). During the process, black carbon is incorporated on the surface of the material itself and is responsible for the blackening of the degraded substrate. The result is the formation of the so-called black crusts (BC) (Wang et al. 2022) (Fig. 1), which not only damage the aesthetics of the cultural heritage in question, but also affect the physical and mechanical properties of the material of which it is made and its durability over time (Pozo-Antonio et al. 2017; Ricciardi et al. 2022a).

In fact, the formation of BCs leads to a detachment of the degraded layer since the formed materials have different texture and porosity respect to the substrate, consequently the adhesion between the BC layer and the substrate is compromised, and so the building material lose its original mechanical properties (La Russa and Ruffolo 2021).

Analyses on the surface of BCs and their main elements are widely reported in the literature (Marinoni et al. 2003; Bonazza et al. 2005; Belfiore et al. 2013; Comite et al. 2017, 2020b, c, 2021; Farkas et al. 2018; Fermo et al. 2018), but there are few studies concerning the presence and origin of organic pollutants such as polycyclic aromatic hydrocarbons (PAHs) on BCs (Gianguzza et al. 2004; Martínez-Arkarazo et al. 2007; Orecchio 2010; Prieto-Taboada et al. 2013; Lamhasni et al. 2019; Islam et al. 2024). BCs, in fact, can be regarded as passive samplers of atmospheric pollutants (metals, PAHs, etc.) in atmospheric particulate matter that are typical of the area in which it is formed (Comite et al. 2017; Fermo et al. 2018; Ricciardi et al. 2022a). Their analysis is therefore very interesting because they can derive from periods of accumulation of pollutants which can also be very long (over 100 years).

PAHs are a class of chemical compounds that consist of at least two annelated aromatic rings and originate from the incomplete combustion of fuels (fossil fuels, coal, and biomasses) and/or emitted during natural processes (e.g., forest fires and volcanic eruptions) (Galmiche et al. 2021; Soursou et al. 2023). They can be divided into subgroups based on the number of aromatic rings and have showed the ability to accumulate in diverse environmental matrices, posing a threat to humans and biota (Soursou et al. 2023). Although more than 100 different PAHs have been identified in the environment, only 16 PAHs have been included in the list of priority contaminants by the United States Environmental Protection Agency due to their toxicity (Mallah et al. 2022). Given their widespread presence in the environment and the problems associated with them, there are several studies in the literature on their quantification in different environmental matrices (air, water, soil, microorganisms, microplastics, etc.) (Kim et al. 2013; Mojiri et al. 2019; Reizer et al. 2022; Chen et al. 2022; Pacín et al. 2023; Sanli et al. 2023). Consequently, considerable analytical efforts have been carried out in order to correctly quantify this class of compounds, as can be seen from the reviews recently reported in the literature (Galmiche et al. 2021; Soursou et al. 2023). Furthermore, another fundamental aspect concerns the identification of the sources of PAHs in the environment, which can be carried out through the analysis of the distribution of the different PAHs, the use of diagnostic ratios, and other statistical analyses (Tobiszewski and Namieśnik 2012).

The aim of this study is the detection and the quantification of PAHs in BC samples from the Monumental Cemetery of Milan (Italy), together with the assessment of their sources through the analysis of their distributions in the samples and the use of diagnostic ratios.

## Materials and methods

### Materials

All the chemicals used for the measurements (PAH calibration mix TraceCERT, certified reference material, 10 μg/mL each component in acetonitrile, dichloromethane, pentane, acetonitrile LC/MS grade, water LC/MS grade) were purchased from Sigma-Aldrich (St. Louis, MO, USA).

### Sampling points

**Fig. 2:**
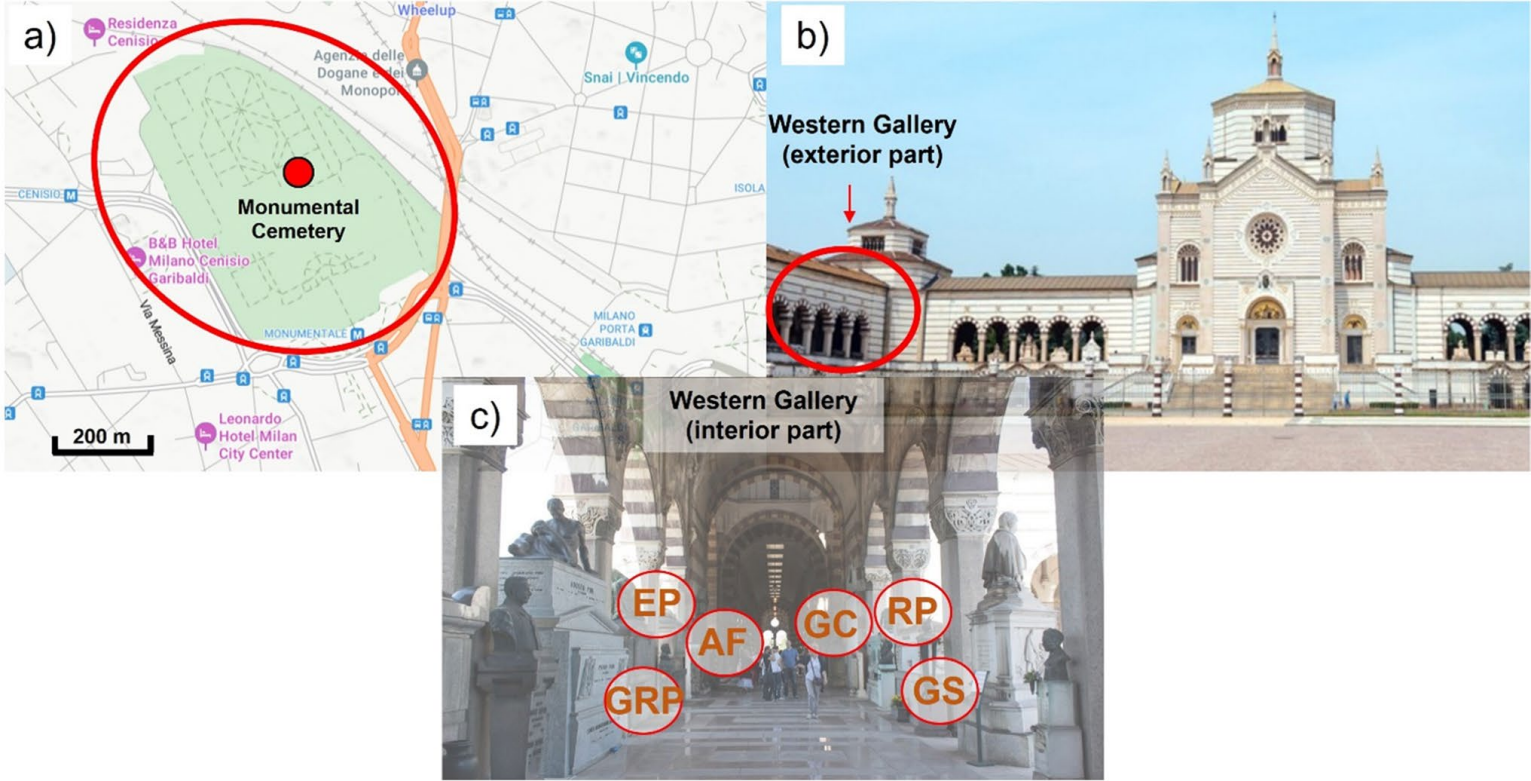
**a** Location of the Monumental Cemetery (red circle) into the Milan city center; **b** Famedio of the Monumental Cemetery with indication of the location of the Western Gallery (red circle); **c** Location of the sampling points inside the Western Gallery

The Monumental Cemetery of Milan (Fig. 2 a and b) is one of the most important and best-known Italian cemeteries and a prominent cultural site in Italy and Europe (Selvafolta 2007a). From the central building, along the east–west axis of the façade, there are symmetrical porticos called *Gallery*, which connect the building orthogonally to the so-called *Edicole* (Selvafolta 2007b). In this study, six fragments fallen from some of the monuments in the Western Gallery (Fig. 1c) were sampled between 2019 (GS, AF, GRP, EP) and 2020 (RP, GC) (Comite et al. 2020a; Ricciardi et al. 2024a). These samples are representative of the different types of black crusts present on sculptures in a very exposed area of this cemetery since it is a semi-confined environment. The sculptures under study are works of art dedicated to the memory of deceased members of wealthy Milanese families, so they do not have proper names, but are identified with an abbreviation containing only the initials of the name and surname of the deceased. The sculptures are located in one of the galleries of the Monumental Cemetery, which are outdoor areas with an arcade structure, mostly protected from rainwater. There are currently no plans to restore these galleries. Two sculptures (AF and GC) are located in a more sheltered area of the Western Gallery, while the other four (GS, GRP, RP, and EP) are in more open parts of the Western Gallery. Most of the sample are taken from marble sculptures, only EP sample comes from a calcarenite sculpture.

About 1 g of each sample (BC with the carbonate matrix) was placed in a plastic bag, and stored refrigerated (4 °C), avoiding the exposure to light, until the analysis was performed. Based on the year of sampling and the year of construction of each grave monument, it is possible to estimate the years of pollutants’ accumulation in the samples (Table 1). However, although there are no historical-artistic indications of previous falls of crusts from the surface of these monuments, these events cannot be ruled out entirely. Therefore, the pollutant accumulation time calculated in this way is to be considered only an estimate, there may be a time bias.

**Table 1:** Description of BC samples in terms of year of pollutants’ accumulation

Sample	Year of monument construction	Year of pollutants’ accumulation*
GS	1870	149
AF	1875	144
GRP	1900	119
RP	1884	136
GC	1906	114
EP	1922	98

*Difference between year of sample collection and year of monument construction

### High-performance liquid chromatography (HPLC) measurements

Before the analysis, BC samples were finely pulverized in a mortar. A Soxhlet extraction was carried out on a weighted amount of sample (in the range 0.2–0.5 g) using a mixture of dichloromethane/pentane:1/1 v/v for 12 h, following a literature procedure (Orecchio 2010). After slow evaporation of the extracting mixture (rotary evaporator with the thermostatic bath at T = 35 ± 0.5 °C until a volume of 3 mL, and dried under a weak nitrogen flow), the obtained extracts were diluted with acetonitrile and passed through a 0.45-μm filter prior to the high-performance liquid chromatography (HPLC) analysis. HPLC measurements were performed using an Ultimate 3000 Thermo Scientific system equipped with a Diode-Array detector (DAD), binary pump, C18 reversed-phase column (Luna 5 μ, 150 mm × 4.6 mm i.d., pore size of 5μm, Phenomenex) and automatic sample injector (loop of 100 μm). A mobile phase made of acetonitrile/water: 70/30 v/v, a flow rate of 1.2 mL/min were used for the chromatographic runs. Chromatograms were recorded at 220, 254, 265, and 280 nm, while all PAHs were quantified using the signal at 280 nm, a wavelength that allows better quantification of those PAHs with lower concentrations in the BC samples by eliminating signal interference. Calibration curves (a total of 5 calibration standards) for PAHs were prepared by diluting a PAH calibration mix (10 μg/mL) with acetonitrile to the range 0.01–1 μg/mL. The identification of PAHs in the sample extracts was performed on the basis of previously determined retention times and confirmed by the analysis of the UV spectra. NP: y = 0.52833x − 0.00256, *R*^2^ = 0.99995; ACY: y = 0.187459x − 0.000318, *R*^2^ = 0.99987; ACE: y = 0.186524x − 0.000216, *R*^2^ = 0.999986; FL: y = 0.63474x − 0.00190, *R*^2^ = 0.99995; PHE: y = 0.623985x − 0.001319, *R*^2^ = 0.999995; ANT: y = 0.99773x − 0.00539, *R*^2^ = 0.99995; FLA: y = 1.441605x − 0.000037, *R*^2^ = 0.999986; PYR: y = 0.38893x − 0.00096, *R*^2^ = 0.99990; CHR: y = 0.98534x − 0.00690, *R*^2^ = 0.99998; BaA: y = 4.308451x − 0.027636, *R*^2^ = 0.999988; BbF: y = 1.61069x − 0.01651, *R*^2^ = 0.99984; BkF: y = 1.29670x − 0.01719, *R*^2^ = 0.99978; BaP: y = 2.18449x − 0.02135, *R*^2^ = 0.99991; IcdP: y = 2.71077x − 0.02839, *R*^2^ = 0.99991.

Limit of detection (LOD) and limit of quantification (LOQ) were calculated based on the standard deviation of the response and the slope, using the following equations:

1 $$LOD=\frac{3.3* \sigma }{S}$$

2 $$LOQ=\frac{10* \sigma }{S}$$

where σ is the standard deviation of the calibration curve and S is the slope of the calibration curve.

LOD and LOQ in solution (μg/mL) were converted into the corresponding limits as μg/g by considering the weight of BC samples and the volume of acetonitrile used (Table 2).

**Table 2:** List of PAHs detected in the black crust samples, with their abbreviation, and limit of detection (LOD) and limit of quantification (LOQ), expressed as μg of compound per g of dry weights

PAH	Abbreviation	Limit of detection (μg/g)	Limit of quantification (μg/g)
Naphthalene	NP	0.042	0.12
Acenaphthylene	ACY	0.026	0.078
Acenaphthene	ACE	0.027	0.081
Fluorene	FL	0.020	0.060
Phenanthrene	PHE	0.015	0.046
Anthracene	ANT	0.025	0.075
Fluoranthene	FLA	0.016	0.048
Pyrene	PYR	0.071	0.022
Chrysene	CHR	0.017	0.051
Benzo[a]anthracene	BaA	0.012	0.037
Benzo[b]fluoranthene	BbF	0.060	0.18
Benzo[k]fluoranthene	BkF	0.059	0.18
Benzo[a]pyrene	BaP	0.033	0.10
Indeno[1,2,3-cd]pyrene	IcdP	0.034	0.10

## Results and discussion

### Concentration and distribution of the different PAHs on the BC samples

The BC samples from the monumental Cemetery of Milan have been already characterized in terms of their main components, as reported in (Comite et al. 2020a). Briefly, scanning electron microscopy/energy-dispersive X-ray spectroscopy and Fourier-transform infrared spectroscopy with attenuated total reflection analyses showed that all the BC samples are made of gypsum, with a small amount of carbonate deriving from the matrix. Obviously signals due to PAH presence cannot be highlighted from FT-IR spectra because of the very low concentration.

The concentrations of PAHs, as μg of compound per g of dry weights, for the different sample are shown in Table 3. Results are given as mean value of triplicate analyses of each sample. A total of 14 PAHs were identified (NP, ACY, ACE, FL, PHE, ANT, FLA, PYR, CHR, BaA, BbF, BkF, BaP, and Icd). Benzo[g,h,i]perylene and dibenz[a,h]anthracene were not detected (concentration < LOD ~ 0.07 ug/g). The total PAHs concentration varied from 0.72 μg/g (GC) to 3.81 μg/g (GS), with a mean of 1.87 μg/g. As expected, the lower concentrations were observed for the BC samples with lower years of pollutants’ accumulation, i.e., GC and EP (Table 3).

**Table 3:** Concentration of each detected PAHs expressed as μg of compound per g of dry weights, with standard error

PAH (μg/g)	Sample						
	GS	AF	GRP	RP	GC	EP	Range
NP	0.22 ± 0.01	0.20 ± 0.01	0.26 ± 0.01	0.16 ± 0.01	0.12 ± 0.01	0.19 ± 0.01	0.12–0.22
ACY	0.20 ± 0.01	0.17 ± 0.01	0.08 ± 0.01	0.11 ± 0.01	N.Q	N.Q	0.08–0.20
ACE	0.16 ± 0.01	0.14 ± 0.01	0.10 ± 0.01	0.09 ± 0.01	N.Q	N.Q	0.09–0.16
FL	0.52 ± 0.01	0.28 ± 0.01	0.31 ± 0.01	0.33 ± 0.01	0.09 ± 0.01	0.06 ± 0.01	0.06–0.52
PHE	0.13 ± 0.01	0.11 ± 0.01	N.D	N.D	0.22 ± 0.01	0.51 ± 0.01	0.11–0.51
ANT	0.39 ± 0.01	0.19 ± 0.01	0.19 ± 0.01	0.16 ± 0.01	0.08 ± 0.01	N.D	0.08–0.39
FLA	0.34 ± 0.03	0.18 ± 0.01	0.06 ± 0.01	0.05 ± 0.01	N.Q	N.Q	0.05–0.34
PYR	0.62 ± 0.01	0.57 ± 0.01	0.34 ± 0.01	N.D	N.Q	N.Q	0.34–0.62
CHR	0.24 ± 0.01	0.17 ± 0.01	0.11 ± 0.01	0.12 ± 0.01	0.05 ± 0.01	N.D	0.05–0.24
BaA	0.14 ± 0.01	0.12 ± 0.01	0.10 ± 0.01	0.08 ± 0.01	0.04 ± 0.01	N.D	0.04–0.14
BbF	0.30 ± 0.01	0.24 ± 0.01	0.17 ± 0.01	N.D	N.D	N.D	0.17–0.30
BkF	0.21 ± 0.01	0.19 ± 0.01	N.D	N.D	N.D	N.D	0.19–0.21
BaP	0.20 ± 0.01	0.15 ± 0.01	0.14 ± 0.01	0.12 ± 0.01	N.Q	N.Q	0.12–0.20
IcdP	0.15 ± 0.01	0.14 ± 0.01	N.D	N.D	0.11 ± 0.01	N.D	0.11–0.15
Total	3.81 ± 0.05	2.86 ± 0.05	1.85 ± 0.04	1.22 ± 0.04	0.72 ± 0.02	0.76 ± 0.03	0.72–3.81

*N.D.* not detected, *N.Q.* not quantified

It is worth to note that total PAH concentrations in this study were higher than the concentrations reported in the Valley of the Temples of Agrigento (range 0.018–0.084 μg/g) (Orecchio 2010), while similar to those observed on Palermo stone monuments (range 0.077–9.8 μg/g) (Gianguzza et al. 2004) and lower than those detected on an historical building in Bilbao (up to 20 μg/g) (Martínez-Arkarazo et al. 2007) and Getxo (0.4–19 μg/g) (Prieto-Taboada et al. 2013). This is indicative of the fact that the Monumental Cemetery of Milan is placed in the city center and is surrounded by high-traffic road arteries, and traffic is one of the main sources of PAHs.

**Fig. 3:**
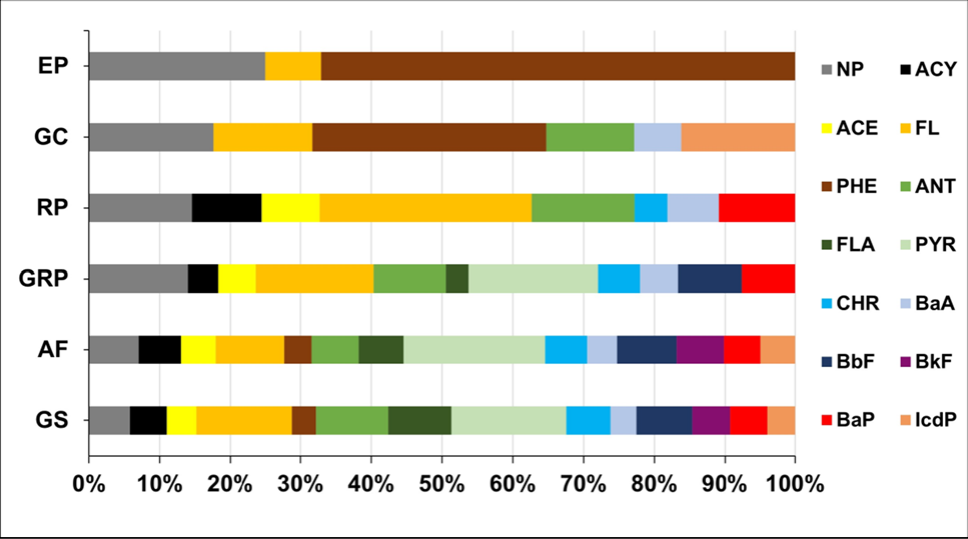
Distribution of the different PAHs on the black crust samples analyzed

Among PAHs, NP and FL were detected in all BC samples (Fig. 3), with concentration ranging from 0.12 to 0.22 μg/g and from 0.06 to 0.52 μg/g, respectively. Concerning the others, ANT and BaA were present in five samples (GS, AF, GRP, RP, and GC), ACY, ACE, PHE, FLA, CHR, BaA, and BaP were observed in four samples, PYR, BbF, and IcdP were found in three samples, while BkF was detected in only two samples.

Ultimately, only for the BC samples with the highest year of pollutants’ accumulation (GS and AF), all the PAHs considered were detected. Moreover, the number of PAHs found in the BCs decreased to three in the sample with the lowest years of pollutants’ accumulation (EP), with a prevalence of low molecular weight PAHs. PYR is the most abundant PAHs in three out of six samples (GS, AF, GRP), whereas FL is the prevalent PAHs in one sample (RP), and PHE in the remaining two samples (GC and EP) at concentration up to 0.62, 0.52, and 0.51 μg/g, respectively. Among known carcinogenic PAHs, BaP accounts for 5–10% of total PAHs in BC samples, with concentration up to 0.20 μg/g.

### Sources of PAHs: diagnostic ratios

**Fig. 4:**
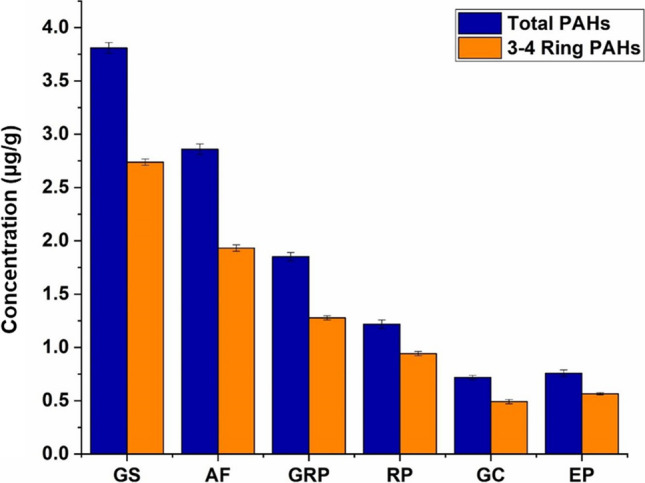
Total PAHs and 3–4 ring PAHs on BC samples

As reported in the literature, it is possible to establish the processes that generate PAHs by studying their distribution in the samples (Tobiszewski and Namieśnik 2012; Wu et al. 2021). Low molecular weight PAHs are generally emitted from low temperature processes (e.g., wood burning), while high molecular weight PAH are mainly formed during high temperature processes, such as the combustion of fuels (Mostert et al. 2010). Moreover, at high temperatures, organic compounds are cracked to reactive radicals, forming stable PAHs (pyrogenic), that are less alkylated and contain more aromatic rings respect to petrogenic PAHs (Hwang et al. 2003). First of all, we can calculate the percentage of PAHs with between 3 and 4 aromatic rings compared to PAHs with more than 4 aromatic rings (Table 4). For all the BC samples under study, most of the PAHs identified fall into the 3–4 aromatic ring category (Fig. 4), with a percentage of 67.5–77.3%. This finding suggests that PAHs in these BC samples mainly derive from high-temperature sources, e.g. combustion of fossil fuels (Orecchio 2010).

**Table 4:** Results of total PAHs (μg/g), PAHs > 4 ring (μg/g) and PAHs 3–4 ring (μg/g and %) with their standard errors, for all black crust samples analyzed

Sample	PAHs total (μg/g)	PAHs > 4 ring (μg/g)	PAHs 3–4 ring (μg/g)	PAHs 3–4 ring (%)
GS	3.81 ± 0.05	0.85 ± 0.02	2.74 ± 0.03	71.8 ± 0.1
AF	2.86 ± 0.05	0.71 ± 0.02	1.93 ± 0.03	67.5 ± 0.1
GRP	1.85 ± 0.04	0.40 ± 0.02	1.28 ± 0.02	69.2 ± 0.2
RP	1.22 ± 0.04	0.20 ± 0.02	0.94 ± 0.02	77.3 ± 0.3
GC	0.72 ± 0.02	0.04 ± 0.01	0.49 ± 0.02	68.7 ± 0.4
EP	0.76 ± 0.03	-	0.57 ± 0.01	74.5 ± 0.3
Mean (Range)	1.87 (0.72–3.81)	0.44 (0.04–0.85)	1.32 (0.49–2.74)	71.5 (67.5–77.3)

To investigate in more detail the sources of PAHs in environmental samples, the so-called diagnostic ratios, i.e. ratios of concentrations of specific PAHs, are extensively used in the literature (Ravindra et al. 2008; Katsoyiannis et al. 2011; Tobiszewski and Namieśnik 2012; Wu et al. 2021). Their application is based on the thermodynamic stability of the compounds considered, in order to draw conclusions on the processes that originated them. Despite their widespread use, their accuracy and reliability in identifying the sources of PAHs has been debated (Katsoyiannis and Breivik 2014; Wu et al. 2021). Indeed, several authors have pointed out criticisms in the use of diagnostic ratios related to differences in gas-particle partitioning behavior and atmospheric persistence of PAHs (Galarneau 2008; Kong et al. 2010; Wu et al. 2021). Consequently, caution must be exercised when using diagnostic reports to derive useful information on sources and patterns of PAHs in the atmosphere. The authors chose to apply the approach reported by (Tobiszewski and Namieśnik 2012) for the BC samples investigated, as it is the most accredited and accepted in the literature.

In the present study, the following six diagnostic ratios were calculated: ANT/(ANT + PHE), BaA/(BaA + CHR), FLA/(FLA + PYR), BaP/(BaP + CHR), FL/(FL + PYR), and PYR/(PYR + BaP). ANT/(ANT + PHE), BaA/(BaA + CHR), and FLA/(FLA + PYR), are used to distinguish between petrogenic (e.g., release of fossil fuels) and pyrogenic (combustion of biomass and fossil-fuels) sources of PAHs (Tobiszewski and Namieśnik 2012). Moreover, in the case of pyrogenic origin, some of them may give further information on which combustion process is prevalent (biomass, petroleum fuels, or coal) (Ravindra et al. 2008). Specifically, ANT/(ANT + PHE) ratio > 0.10 is taken as an indication of high temperature processes (combustion), whereas a ratio < 0.10 indicates low-temperature processes; FLA/(FLA + PYR) ratio < 0.4 and > 0.5 suggests petroleum input and grass, wood, or coal combustion respectively, while ratio between 0.4 and 0.5 indicates liquid fossil fuel combustion; BaA/(BaA + CHR) ratio < 0.2 was indicative of petroleum origin, ratio > 0.35 of vehicular emission. Finally, with the diagnostic ratios BaP/(BaP + CHR), FL/(FL + PYR), and PYR/(PYR + BaP), it is possible to estimate the contribution of diesel or gasoline engines on PAH emissions (Cerqueira and Matos 2019): FL/(FL + PYR) ratio < 0.5 indicates gasoline emission, while PYR/(PYR + BaP) ratio > 0.7 suggests diesel emission.

The calculated diagnostic ratios for BC samples under study are reported in Table 5. Certain values could not be calculated because concentrations below the limit of quantification were observed for at least one of the PAHs under consideration (for EP none of the diagnostic reports could be calculated).

**Table 5:** Main diagnostic ratios for all black crust samples analyzed

Diagnostic ratio	Sample				
	GS	AF	GRP	RP	GC
ANT/(ANT + PHE)	0.75	0.63	NC	NC	0.27
BaA/(BaA + CHR)	0.37	0.43	0.46	0.42	0.46
FLA/(FLA + PYR)	0.35	0.24	0.14	NC	NC
BaP/(BaP + CHR)	0.46	0.48	0.54	0.50	NC
FL/(FL + PYR)	0.46	0.33	0.48	NC	NC
PYR/(PYR + BaP)	0.75	0.79	0.71	NC	NC

*NC* not calculated

The ratio ANT/(ANT + PHE) observed for all the samples is > 0.10 for all the samples, indicating a prevalence of combustion processes. In the case of the BaA/(BaA + CHR) ratio, values in the range 0.37–0.46 were obtained, giving more detail on the type of pyrogenic source as vehicular emission (ratio > 0.35). On the contrary, the calculated FLA/(FLA + PYR) ratio would seem to indicate a petrogenic origin (ratio < 0.4). Concerning the last three ratios, it is not possible to identify a clear preponderance between diesel and gasoline emissions. In fact, the BaP/(BaP + CHR) ratio gives values around the discriminatory threshold of 0.5, while FL/(FL + PYR) would indicate gasoline emission (ratio < 0.5) and PYR/(PYR + BaP), on the contrary, diesel emission (ratio > 0.7). This result can be easily explained if we consider the long accumulation times of pollutants for the samples under examination and the use of both types of fuels in engines in Italy.

### Correlation of total PAHs with elemental carbon and year of pollutants’ accumulation

The literature also reports possible correlations between PAH concentrations and those of other types of pollutants, both organic (organic carbon, elemental carbon) (Crimmins et al. 2004; Arnott et al. 2005; Li et al. 2009) and inorganic (Orecchio 2010; Bernalte et al. 2012), found in particulate matter and/or soil (Rajput et al. 2013). Close correlations between the concentrations of PAHs and elemental carbon (EC) have been ascribed to co-emission, co-transport, and sorption of them (Han et al. 2015). Significant correlations were found both when EC and PAHs originated mainly from fossil fuel (Crimmins et al. 2004; Arnott et al. 2005) and biomass combustion (Li et al. 2009). Furthermore, an association between the concentration of PAHs and organic carbon (OC) is considered to dominate in remote areas, where there is greater transport of these pollutants, while the correlation with EC is prevalent in areas closer to the emission sources (Nam et al. 2008).

In Table 6, the concentration (wt%) of OC and EC and the ratios EC/OC and EC/TC (total carbon, referred as the sum of EC and OC) for EP, GRP, AF, and GS are reported. These values were previously measured on these samples by an innovative analytical method based on thermal analysis (thermogravimetric and differential scanning calorimetry analyses) in a range between 30 and 800 °C, increasing the temperature at 20 °C/min both in inert and oxidizing atmosphere (Comite et al. 2020a).

**Table 6:** OC (wt%), EC (wt%), ratios EC/OC, and EC/TC of some BC samples

Sample	OC (wt%)	EC (wt%)	EC/OC	EC/TC
EP	1.38 ± 0.02	0.44 ± 0.01	0.32 ± 0.03	0.24 ± 0.02
GRP	0.38 ± 0.01	0.35 ± 0.02	0.92 ± 0.05	0.48 ± 0.04
AF	0.30 ± 0.01	0.54 ± 0.03	1.78 ± 0.07	0.64 ± 0.06
GS	0.63 ± 0.01	1.36 ± 0.05	2.17 ± 0.08	0.68 ± 0.06

Data from (Comite et al. 2020a)

**Fig. 5:**
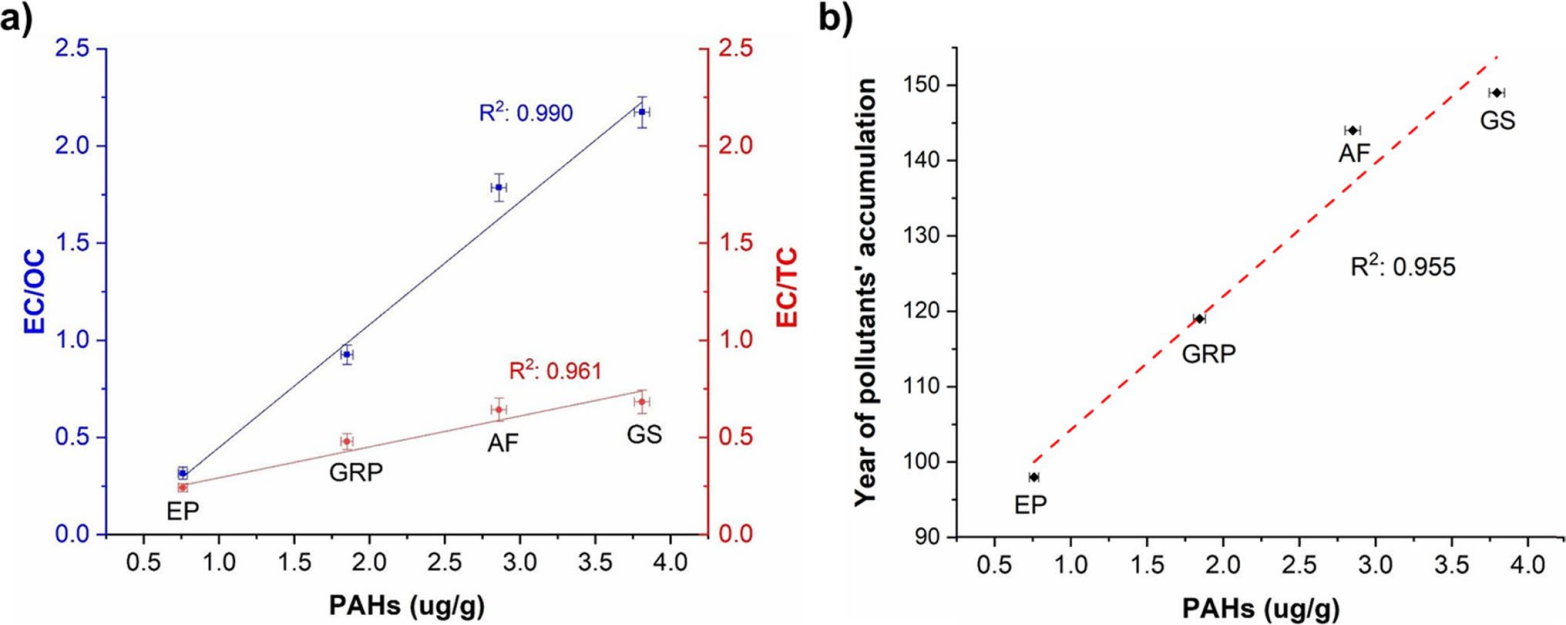
Correlation graph between total PAHs concentration (μg/g) and the ratio EC/OC and EC/TC (**a**) and years of pollutants’ accumulation (**b**)

No significant correlations (at the 0.05 level) were observed between total PAHs concentration (ug/g) and absolute values of EC and OC, with *R*^2^ of 0.64 and 0.40 respectively. On the contrary, significant positive correlation coefficients (at the 0.05 level) are found between total PAHs (ug/g) and EC/OC ratio (Pearson’s* r* of 0.995 and *R*^2^ of 0.990), and between total PAHs (ug/g) and EC/TC ratio (Pearson’s *r* of 0.980, *R*^2^ of 0.961), as can be viewed in Fig. 5a. A preponderance of elemental carbon over organic carbon occurs with accumulation time of pollutants, probably due to the less stability of the organic carbon fraction. Since the EC/OC ratio increases as the years of pollutants’ accumulation in the BCs increase, the same occurs for total PAHs. In fact, a significant strong correlation was obtained between total PAH concentration and the age of the BCs (Pearson’s *r* of 0.977 and *R*^2^ of 0.955, see Fig. 5b).

Although based on a small number of samples, these results further confirm that the predominant origin of the PAHs in the BC samples under study is the combustion of fossil fuels. Indeed, high EC/OC ratios are often associated with primary particulate matter, which is emitted by combustion processes and traffic.

Ultimately, the analysis of the BC samples from the Monumental Cemetery of Milan showed that over a time span of 100–150 years there was a non-negligible accumulation of PAHs on the monuments considered. This is an important aspect to consider since a recently published study shows that black crusts deposited on historical monuments can pose a health risk due to air pollution of the surrounding environment, especially if appropriate precautions are not taken (Islam et al. 2024). Moreover, the study of the distribution and diagnostic ratios, together with other data already reported in the literature (EC and OC), allowed us to confirm that the presence of anthropogenic sources such as traffic and the proximity of the train station is the major cause of the degradation of the monuments contained in this Cemetery.

## Conclusions

In this study, PAHs were detected and quantified in six BC samples from the Monumental Cemetery of Milan (Italy): 14 PAHs were identified (NP, ACY, ACE, FL, PHE, ANT, FLA, PYR, CHR, BaA, BbF, BkF, BaP, and IcdP). The total PAH concentration varied from 0.72 μg/g (GC) to 3.81 μg/g (GS), with a mean of 1.87 μg/g. Since these BC samples come from sculptures located in the same part of the Monumental Cemetery (Western Gallery), exposed to almost the same degree of pollution, the observed differences in PAH abundance can be ascribed to the different accumulation times of the pollutants. In fact, the lower concentrations were observed for the BC samples with lower years of pollutants’ accumulation. PYR is the most abundant PAHs in three out of six samples (GS, AF, GRP), whereas FL is the prevalent PAHs in one sample (RP), and PHE in the remaining two samples (GC and EP) at concentration up to 0.62, 0.52, and 0.51 μg/g, respectively. Among known carcinogenic PAHs, BaP account for 5–10% of total PAHs in BC samples, with concentration up to 0.20 μg/g.

For all the BC samples under study, most of the PAHs identified fall into the 3–4 aromatic ring category (67.5–77.3%). This result suggests that PAHs mainly derive from high temperature sources (e.g., combustion of fossil fuels). The diagnostic ratios ANT/(ANT + PHE) and BaA/(BaA + CHR) indicate a prevalent origin from combustion processes, such as vehicular emission. Moreover, it is not possible to identify a clear preponderance between diesel and gasoline emissions since the diagnostic ratios BaP/(BaP + CHR), FL/(FL + PYR), and PYR/(PYR + BaP) give mixed results. This can be easily explained if we consider the long accumulation times of pollutants (100–150 years) for these BCs and the use of both types of fuels in engines in Italy.

Finally, the analysis of the BC samples from the Monumental Cemetery of Milan showed that over a time span of 100–150 years there was a non-negligible accumulation of PAHs on the monuments considered. Furthermore, the analysis of the distribution of the different PAHs and the diagnostic ratios, together with correlation with EC/TC, allowed us to confirm that anthropogenic sources such as traffic are the major cause of the degradation of the monuments contained in this cemetery.

## Data Availability

Not applicable.
